# Strangulated internal supravesical hernia associated with left inguinal hernia: A very rare case report of acute intestinal obstruction

**DOI:** 10.1016/j.amsu.2021.102393

**Published:** 2021-05-13

**Authors:** Othmane Elyamine, Fatimazahra Bensardi, Abdessamad majd, El Bakouri Abdelilah, Bouali Mounir, El Hattabi Khalid, Fadil Abdelaziz

**Affiliations:** a Visceral Surgery Emergency Department, University Hospital Center Ibn Rochd, Casablanca, Morocco; b Faculty of Medicine and Pharmacy, Hassan II University, Casablanca, Morocco

**Keywords:** Bowel obstruction, Internal supravesical, Inguinal hernia

## Abstract

Internal hernias are a rare cause of acute intestinal obstruction. Supravesical hernia is an exceptional form of hernia that is often diagnosed intraoperatively. The abdominal CT scan performed in the emergency guides the preoperative diagnosis. Our work concerns a patient admitted to the visceral surgical department of the university hospital ibn Rochd of Casablanca for an occlusion dating back to 05 days, the abdmonial x-ray objectified hydro-aerial levels in the small intestine, and the CT scan found an invagination at the left fossa iliaca associated with a double hernia: internal supravesical and left inguinal. The patient was then operated after conditioning and the diagnosis of an internal supravesical hernia was retained per operatively. Internal supravesical hernia is a very rare cause of acute intestinal occlusion and is often diagnosed at the time of surgical exploration.

## Highlights

• Supravesical hernias are rare but potential causes of intestinal.
• Internal hernia of the supravesical fossa is an unusual cause of small bowel obstruction.
• Pre-operative diagnosis of this condition is very difficult.
• The surgical management can be conducted indiscriminately by laparoscopy or laparotomy.
• The treatment is release of the intestinal obstruction and closing the hernial defect to prevent future recurrences.

## 1 Introduction

Described for the first time in 1804 by Sir Astley Cooper [1], supravesical hernias are among the rarest of internal hernias, they are often found in men after the age of 50.

These are almost always acquired hernias, associated in a non-exceptional way with inguinal hernias.

They develop in the supravesical fossa, with the orifice of entrance located between the urachus and the residual fibrous cords of the umbilical arteries. Herniated loops expand into the Retzius space.

This work has been reported in line with the SCARE 2020 criteria [2].

## 2 Case presentation

We report the case of Mr. B. H, 33 years old, without any pathological history admitted for an obstructive syndrome, made of a bowel blockage with vomiting, abdominal pain without externalized digestive hemorrhage, or fever.

The clinical examination found a conscious patient, hemodynamically stable, BP: 12/07, respiratory rate of 17 CPM, Heart Rate: 81 bpm, Temperature: 37.3 C°.

The abdominal examination noted a slightly distended abdomen, tympanic to percussion; a reducible left inguinal hernia, impulsive to cough with a 15 mm collar.

The digital rectal exam found a normal anal margin, good sphincter tone, empty rectal ampulla. The rest of the somatic examination was without peculiarities.

**Fig. 1:**
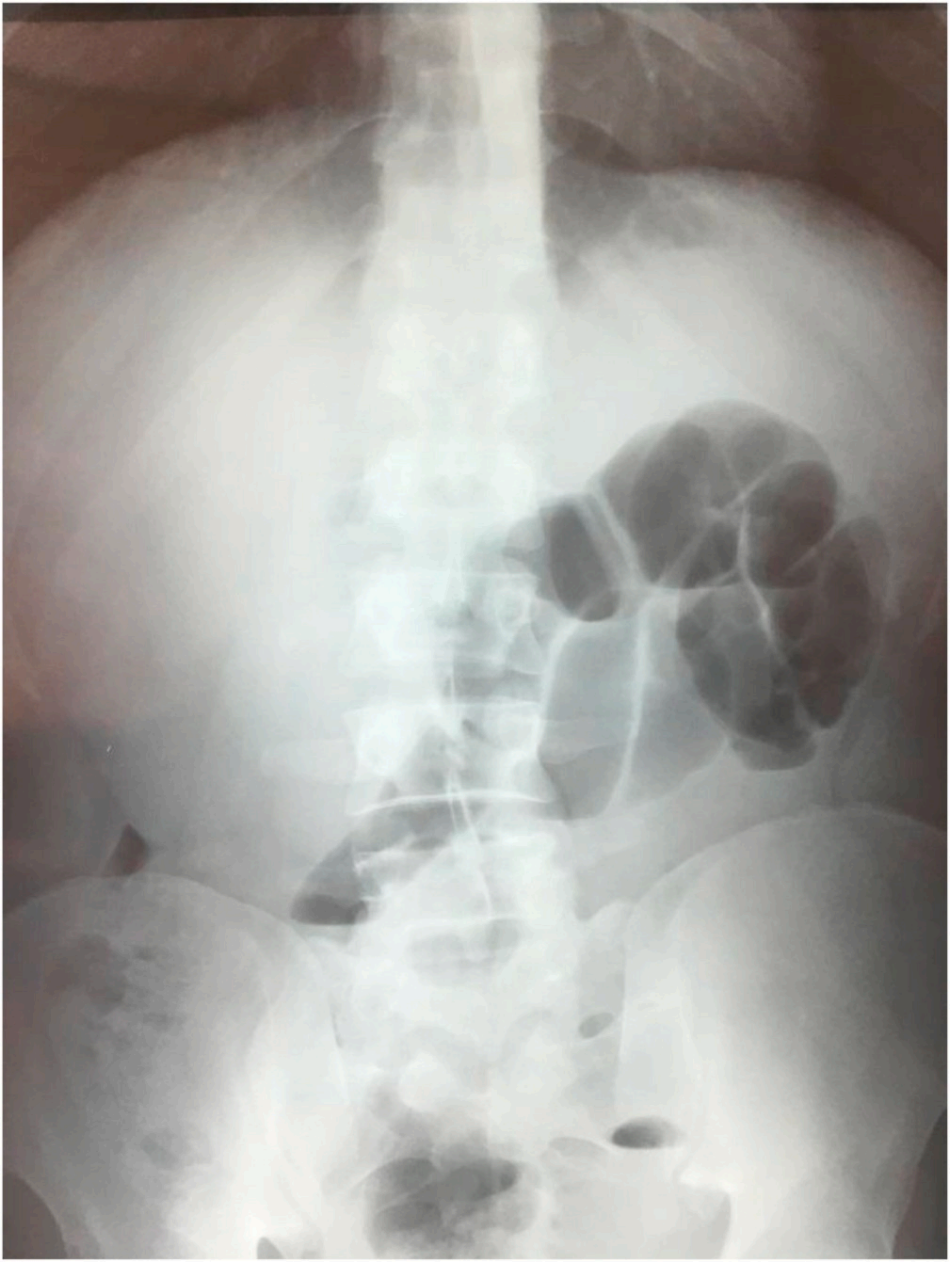
X-ray of the abdomen without preparation showing hail water levels.

In the paraclinical assessment, the X-ray of the abdomen showed hydro aerial levels located in the small intestine **(**Fig. 1**).**

**Fig. 2:**
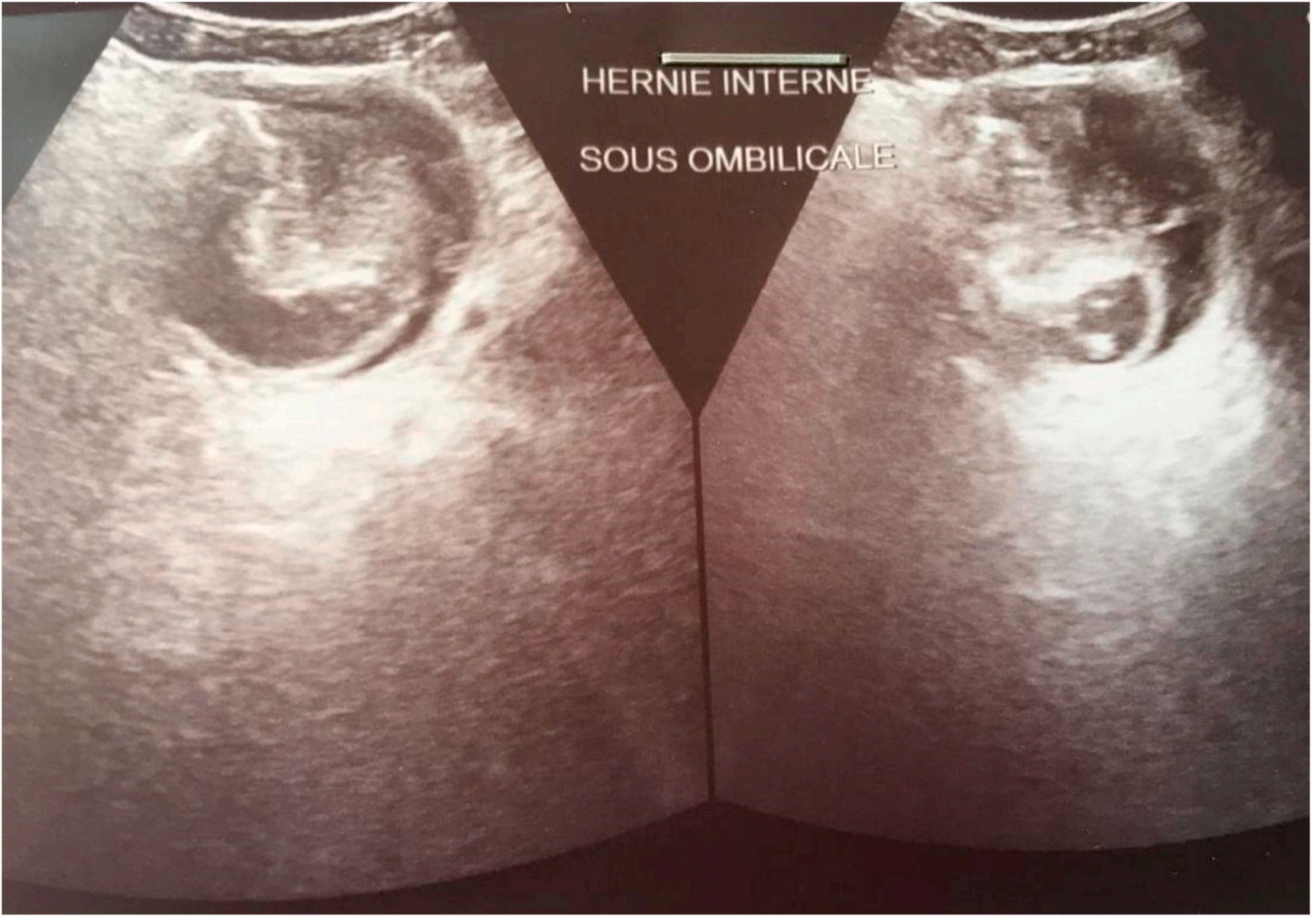
Abdominal ultrasound showing a strangulated internal hernia.

Abdominal ultrasonography found small loops trapped in a sack located four finger spans under the umbilicus and four-finger spans above the pubis in favor of a strangulated internal hernia **(**Fig. 2**).**

**Fig. 3:**
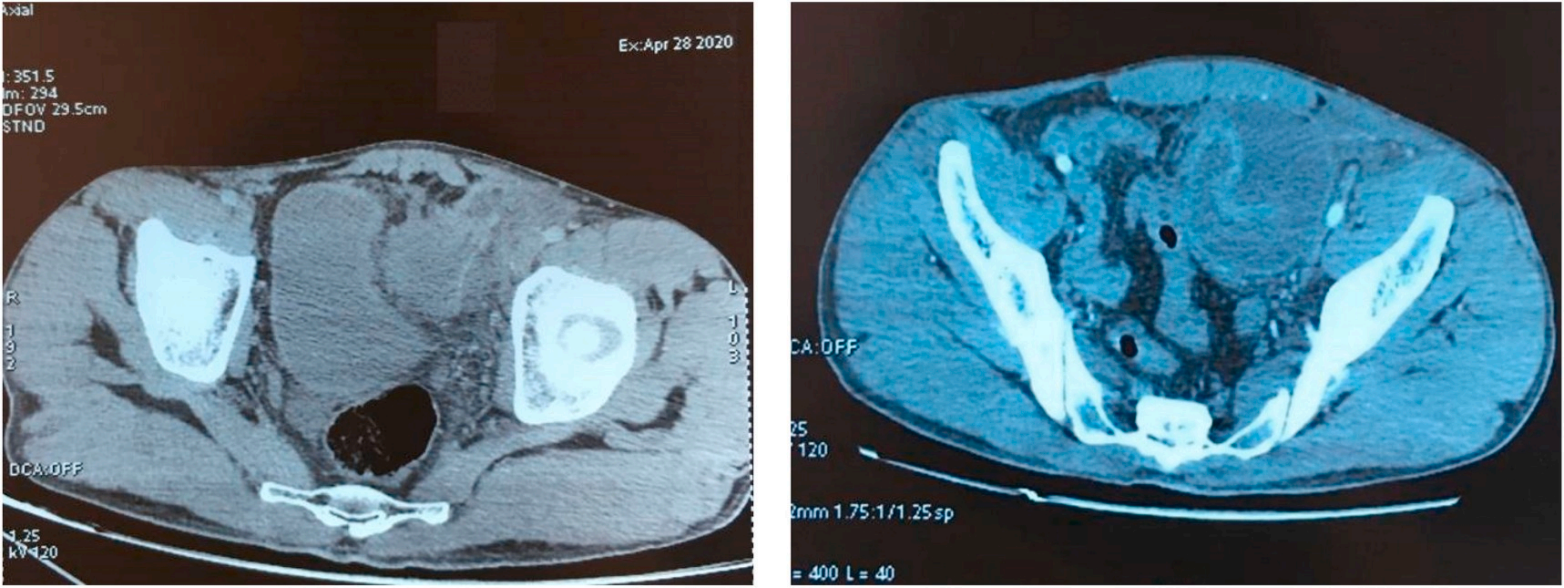
Abdomino pelvic CT scan objectivizing haemophilic invagination at the FIG level associated with a double hernia: internal supra bladder and left inguinal.

Abdomino-pelvic CT scan objectified the presence, in the left fossa iliaca, of a small bowel-like digestive structure that seems to invaginate within another small intestine-like loop, giving the appearance of a spiral tower containing hydro aerial levels measuring 73*68 mm. The distal part of this loop extends up to the inguinal orifice where it herniates in association with epiploid material with no sign of strangulation and with a collar measuring 33 mm. A low abundance of intraperitoneal effusion in the left fossa iliaca was found without parietal pneumatosis **(**Fig. 3**).**

In total: A jejuno-jejunal intussusception in the left fossa iliaca associated with a double hernia: internal supravesical and left inguinal.(Table 1)

**Table 1:** The biological blood test was as followed.

TEST	RESULT
Hemoglobin	14,5 g/dl
White cell count	20980/mm3
Urea	0,48 g/l
Creatinine	7,8 mg/l
Sodium	136 mmol/l
Potassium	3.5 mmol/l
Aspartate transaminase	10 IU/l
C-reactive protein	291.2 mg/l
Albumin	32 g/l
TP	72%

**Fig. 4:**
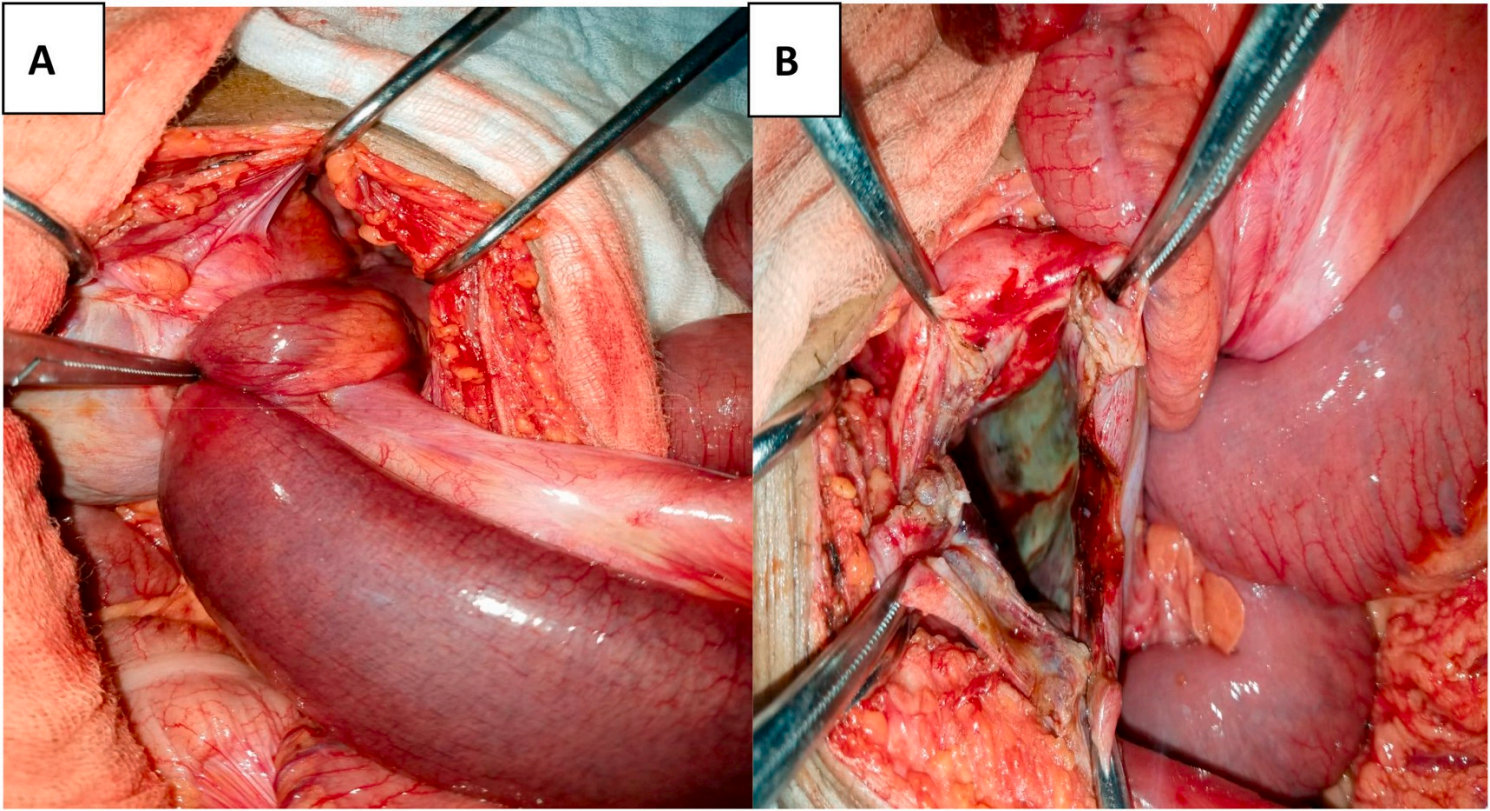
Intraoperative images: A. Incarceration of a small loop in an abnormal orifice of the pelvic parietal peritoneum B. Hernial orifice at the supravesical fossa.

A median laparotomy, above and below the umbilicus, was performed with the following findings: a strangulated internal hernia in the supravesical fossa responsible for jejunal incarceration located at 3 m of the duodenojejunal angle and 70 cm of the ileocecal junction (Fig. 4A) responsible for intestinal distension onward with necrotic content, perforated and with some false membranes located in the hernial sack with effusion.

The patient underwent a 20 cm jejunal resection at 3 m of the duodenal flexure with an end to end jejuno-jejunal anastomosis using interrupted sutures with vicryl 3/0; A partial resection of the hernial sack (Fig. 4B) and its simple closure with vicryl 2/0 in separate stitches was performed, followed by a cure of the left inguinal hernia by raphia.

The postoperative follow-up was simple; the patient was discharged after the sixth day with one year's follow-up.

The anatomopathological study of the surgical specimen showed a necrotico-hemorrhagic ischemia of the jejunal wall, one limit was lesional while the other one was congestive with an absence of neoplasia.

## 3 Discussion

Internal hernias represent only 0.5–1% of all causes of bowel obstruction. A Supravesical internal hernia is an exceptional form of internal hernia and its incidence is difficult to assess [[1], [2], [3], [4], [5], [6]]. Supravesical hernias originate at the expense of the supravesical fossa, which is located between the remnants of the urachus and the umbilical arteries [7,10]. They are formed medially to the umbilical arteries and most often emerge in the Retzius space where they engage laterally in the anterior abdominal wall, thus forming external supravesical or laterovesical hernias [4,9,10]. More rarely, the sac is located around the space surrounding the bladder thus forming the internal supravesical hernia [5,10]. It is more common in men over the age of 50 [4,9].

Diagnosis of acute intestinal occlusion is easy, however, supravesical hernia is an intraoperative finding for many authors [1,4,8,9,11]. Although the preoperative diagnosis remains unusual, some authors had reported cases where the diagnosis was already evoked by abdominal CT scan before surgery as was in our patient's case [1,3,5]. Simple suturing of the hernia sac is sufficient for some authors and prevents recurrence [1,7]. In our case, like some authors [5,7], we performed a 20 cm jejunal resection of necrotic loops with an end to end anastomosis with resection of the excess sac.

Internal supravesical hernias have a good prognosis [1] and depend mainly on early diagnosis and management of bowel obstruction.

## 4 Conclusion

Internal supravesical hernia is a rare pathology. It is most often diagnosed intraoperatively during the management of acute intestinal obstruction. Its surgical treatment is simple by closing the sac to prevent recurrences. In the case of acute intestinal obstruction, it should be kept in mind that an internal supravesical hernia may be an unusual cause.

## Ethics approval

No ethical approval necessary.

## Consent of the patient

Written informed consent was obtained from the patient for publication of this case report and accompanying images. A copy of the written consent is available for review by the Editor-in-Chief of this journal on request.

## Funding

The author(s) received no financial support for the research, authorship and/or publication of this article.

## Provenance and peer review

Not commissioned, externally peer-reviewed.

## Declaration of competing interest

The authors declared no potential conflicts of interests with respect to research, authorship and/or publication of the article.

## Author statement

El yamine othmane: Corresponding author writing the paper and operating surgeon.

Bensardi Fatimazahra: writing the paper and operating surgeon.

Abdessamad majd: writing the paper.

El Bakouri Abdelilah: study concept.

Bouali Mounir: writing the paper.

El Hattabi Khalid: study concept.

Fadil Abdelaziz: correction of the paper.
